# Mass tourism urban destinations and climate change in small islands: resilience to extreme rainfall in the Canary Islands

**DOI:** 10.1007/s10668-023-03406-7

**Published:** 2023-06-05

**Authors:** Pablo Ley Bosch, Óscar de Castro González, Francisco García Sánchez

**Affiliations:** 1 Departamento de Arte, Ciudad y Territorio, Universidad de Las Palmas de Gran Canaria, Campus Universitario de Tafira, 35017 Las Palmas de Gran Canaria, Spain; 2 Departamento de Geografía, Urbanismo y Ordenación del Territorio, Universidad de Cantabria, Av. de los Castros, s/n, 39005 Santander, Spain

**Keywords:** Mass tourism, Canary Islands, Climate change adaptation, Extreme rainfall, Vulnerability, Urban planning

## Abstract

The Canary Islands are one of the main destinations for mass tourism in the European context, characterized by the absence of seasonality in tourist activity. Moreover, the level of activity increases during the winters, coinciding with a greater probability of extreme rainfall events, whose danger seems to be increasing as a result of climate change. Owing to its pronounced orography, the southern coast of the island of Gran Canaria houses several tourist settlements built along ravines and steeply sloping terrain. This scenario presents considerable risk because of spatial probability of landslide occurrence. The case of San Agustín, especially, serves to test the model of tourist urbanization along the hillside, demonstrating its high fragility in the face of extreme rainfall events. Especially owing to its importance in providing assistance in emergency situations, its vulnerability has been analyzed with regard to accessibility, which is entirely dependent on road mobility. The growth model of San Agustín serves as an example of mass tourism in small islands, allowing urban planners and designers to assess corrective measures based on managing its existing road infrastructure and open spaces right from the planning stage.

## Introduction

Mass tourism as a globalized consumption model has been defined as the movement of a large number of tourists to popular holiday destinations (Butler, 1990; Dehoorne et al., 2014). The democratization of travel through the diversification of transport options and the creation of new online tools for receiving tourism services has favored a greater heterogeneity, facilitating easy access to these services (Vainikka, 2013; Gilmore, 2017; Gutierrez et al., 2017). The diversity of mass tourism is also evident from the spatial and functional distribution of tourist cities, which have been increasing their level of complexity in terms of development and management (Fistola & La Rocca, 2017), as well as in terms of greater diversification of the accommodation typologies on offer (Gutiérrez et al., 2017).

The regional and urban expansion of mass tourism has often generated unsustainable urbanization models, to which the distorting phenomenon of climate change has now been added (IPCC, 2018; Scott, 2021). The increase in average temperatures is modifying climate variables with changes in rainfall patterns and the recurrence of extreme climate events (IPCC, 2021). This makes tourist areas subject to hitherto unknown stressors (Mycoo et al., 2022). Risk management requires the coordination of both mitigation and adaptation actions, among them, the improvement of green infrastructure and the promotion of coordination policies between tourism and urban planning (Lopes et al., 2022). The need to strengthen the capacity of tourist destinations to adapt to climate change has been endorsed by several authors (Dłużewska & Giampiccoli, 2021; Moore, 2015; Scott, 2021), as well as international organizations and entities (OMT- Organización Mundial del Turismo, 2007; OECD-UNEP, 2011; UNWTO, 2007, 2018). In this sense, tourism infrastructure, often located along coastal areas, must undergo a serious reconversion toward more sustainable and resilient forms in the face of climate change.

In the specific case of archipelagos and small islands, the scarcity of space available for the expansion of tourist settlements increases the complexity of this fundamental problem (Larjosto, 2020; UNWTO, 2018). Island territories usually offer favorable environments for developing areas intended for mass tourism, giving them greater competitiveness at the global level with their peculiar geographical characterization and environmental qualities, their idyllic image, as well as the availability of a coastline offering the sun, sea and sand combination (Dłużewska & Giampiccoli, 2021). But the rise in sea levels, the constant increase in flood events and urban heat islands in coastal tourist areas highlight the vulnerability of this powerful productive sector to climate change (Moore, 2015; Raven et al., 2018; Dogru et al., 2019; IPCC, 2021; Scott, 2021). In non-sovereign islands and archipelagos, as well as in much of the Small Island Developing States (SIDS), the tourism sector is one of the main economic drivers (Polman et al., 2016; Scheyvens & Momsen, 2008; Thomas et al., 2020). Prior to the COVID-19 pandemic, according to the World Travel & Tourism Council, while the contribution of tourism to the gross domestic product (GDP) of the 10 most visited countries in the world barely exceeded 12%, that in the SIDS averaged at 27%; with even more exceptional figures for destinations such as Maldives (52%), St. Lucia (68%) and Aruba (69%) (WTTC, 2021). In 15 Caribbean countries highly dependent on tourism, Cevik and Ghazanchyan (2020) research studies have indicated an average reduction of 10 percentage points in tourism revenue as a share of GDP due to climate vulnerability.

The scientific community, including a good share of decision makers, is aware of the difficulties facing the tourism sector in this new scenario. There is very high certainty that sunny, seaside destinations, particularly from tropical and subtropical regions, will be affected by climate change (IPCC, 2018, p. 243; IPCC, 2021). However, the impact of the same on such tourist locations is yet to be fully understood. Climate change vulnerability and risk analyses must be adapted to the specificities offered by tourist destinations (Student et al., 2020). Unlike conventional cities settled throughout history, island vacation spots have generally sprung up rapidly on virgin environments, disregarding the knowledge offered by experience. Hence, such destinations tend to be located in areas that are easily susceptible to unforeseen and extreme weather events (Kaján & Saarinen, 2013; Scott, 2021; UNWTO-UNEP, 2008).

The literature review shows various approaches depending on the planning strategies of the tourism sector. Some studies have focused on the direct impacts of climate change on this sector. Wolf et al. (2021) provide evidences on how Tonga and the Solomon Islands have seen reduced touristic attraction due to climate change. The difficulties of managing climate change at tourist destinations are a recurring problem. In Cayman Islands, for instance, the public administration has not yet developed adaptation strategies for its tourism infrastructures despite being aware of the possible impacts (Johnston & Cooper, 2022). Many island destinations are highly dependent on external investors who dominate the tourism sector, such as the Bahamas, which considerably limits the possibilities of adaptation to climate change (Petzold et al., 2018).

A greater concern has been detected in other studies, which present adaptation strategies to climate change (Dłużewska & Giampiccoli, 2021; Larjosto, 2020) as well as the proper management of more resilient development (Aretano et al., 2013; Moore, 2015; Pons & Rullan, 2014; Wardekker, 2021). Such developments need the combination of mitigation and adaptation actions as those identified by Dodds and Kelman (2008) in Magaluf (Mallorca, Balearic Islands) fifteen years ago. An analysis of the climate–tourism relationship in various national policy documents of more than sixty countries has found that just over 30% of the cases studied had progressed toward this necessary integration (Becken et al., 2020). The successful use of strategic planning tools such as the Vanuatu Tourist Adaptation System has made it possible to test adaptation measures for resilient developments (Loehr, 2019). Samoa has illustrated the benefits of mainstreaming climate adaptation actions by getting resilient communities to address climate-sensitive management plans in five tourism development areas (UNWTO, 2018). However, new variables must be incorporated into vulnerability and risk analyses, where the scenarios allow more dynamic development strategies to be proposed. This is the case of Barbados and Curaçao developed by Student et al. (2020), where the dynamic vulnerability experiment allowed an assessment of the risk to sea level rise.

Despite the profusion of literature on climate risk management in conventional cities and urban areas, the specificity of tourist areas has not been adequately studied, especially the particularities of island regions under the umbrella of mass tourism. To address this gap, the present study explores the possible impacts of climate change on tourist settlements and/or small islands with an abrupt orography, vulnerable to extreme rainfall events. The aim of the study is to identify the strategies needed for developing long term, climate resilient urban plans and improving the condition of existing tourist settlements.

## Study area in the context of climate change

### Canary Islands and effects of extreme rainfall on mass tourism

Mass tourism is one of the main economic resources of the Canary Islands. Before the COVID-19 pandemic, the revenue share of tourism in Spain had reached 12.4% of its GDP, generating 12.9% of total employment (INE, 2020). In the Canary Islands, the impact of tourism is much greater (35.0% of GDP), generating 40.4% of total employment opportunities (Exceltur, 2019), which is the highest in the country.

With the arrival of the first waves of mass tourism in the 1960s, the urban expansion associated with this demand has remained constant, favored by speculative processes and lax legislative frameworks that have led to the overexpansion and obsolescence of a good part of tourist settlements developed in the Canaries (Bianchi, 2004; Cáceres & Millán, 2002; Llorca, 2009; Oreja et al., 2008). In 2019, accommodation options in the Canary Islands stood at around 415,000 hotel and non-hotel beds (ISTAC, 2021), including renovated tourist complexes with the highest level of obsolescence.

As in other Spanish regions, the mass tourism destinations in the Canary Islands show a certain fragility in the face of climate change risks (Campos & Puig, 2017; Carrillo et al., 2022). The Canary Islands lie in the outermost periphery of the European Union (EU), on which the islands are dependent to a large extent. The region is also very dependent on the multinationals of the tourism industry (Cáceres & Millán, 2002). Characterized by real estate development mainly funded by foreign capital that hinders the incorporation of generalized adaptation measures against climate change, Petzol et al. (2018) point out that the scenario here is similar to that in the Bahamas. Most of the tourist complexes at the Canary Islands lie near the coastline, taking advantage of the excellent beaches. But sea level rise and storm surge can compromise tens of kilometers along this coastline, affecting urbanized locations and areas with high environmental values (Fraile et al., 2014; Losada et al., 2014; Yanes et al., 2021). In addition, the increase in temperature and heat waves can modify the balance in the availability of water resources, compromise the climatic excellence of the islands and increase the health problems of the population (Carrillo et al., 2022). In particular, atmospheric changes also predispose certain areas to receive previously unrecorded winds and heavy rains (Carrillo et al., 2022; Dorta et al., 2020).

In this respect, rainfall of high hourly intensity has been classified as one of the main threats causing damage to buildings, road cuts or variations in the islands’ coastal profile, closely related to changes induced by urbanization (Dorta, 2007; Dorta et al., 2020; López et al., 2019; Olcina, 2008). Between 1994 and 2020, a total of 59 extreme weather events related to rainfall and floods have been recorded in the Canary Islands (CCS, 2021). With a climate dotted by winter advections of polar air and invasions of Saharan air, the high altitude of the islands favors the appearance of localized and intense rains (Olcina, 2008, p. 51). In urbanized sectors to the south of the islands, the high slope and lack of adequate drainage infrastructure highlight their high vulnerability to landslides (Dorta et al., 2020; López et al., 2019).

The risks introduced by the tourist-led transformation of the natural landscape are not easily predictable. A clear example in south Gran Canaria is the tourist area of Maspalomas where the average annual rainfall of 120 mm in 1950 exceeded to 201.3 mm, a figure that with the current level of urban development would cause severe damage (Dorta, 2007). Although temperatures are very mild throughout the year, extreme rainfall can occur during autumn and winter (AEMET, 2022; Carrillo et al., 2022). During this period, the increase in the number of tourists over the summers usually oscillates around 18–20% (INE, 2020), coinciding with the greatest probability of very heavy precipitation. Floods such as the one that occurred in Gran Canaria in February 1989, or in November 2001 when more than 300 tourists were evacuated, have become random incidents in recent years (AEMET, 2022). Most significant was Cyclone Delta in November 2005, which was an unknown phenomenon with a previously unpredictable level of risk (Dorta, 2007). The most recent incident was the heavy rainfall of September 18, 2022, followed just a week later by the cyclone Hermine, which had converted into a tropical storm that for the first time in the history of the Canary Islands forced it to activate insular emergency plans (PEIN) across the seven islands, putting them simultaneously under maximum alert. Although the PEIN of Gran Canaria does not propose specific measures for tourist areas (PEIN GC, 2005), Hermine brought heavy rains with significant landslides, road closures and cancelation of flights, which especially affected the islands’ tourism sector precisely during its busiest season.

Although climate scenarios predict a generalized shortage of annual rainfall in the Canary Islands, depending on various factors (AEMET, 2021), some research indicates an increase in very intense and localized rainfall phenomena (Díez et al., 2019; Máyer et al., 2017; Tarife et al., 2012). Having become a recurring theme in the current context of climate change, this climatic behavior of extreme rainfall with episodes of floods and landslides, undoubtedly, carries special significance for tourist development in the Canary Islands (Díez et al., 2019).

### San Agustín: a repeated urbanization model of tourist settlement along hillsides

Gran Canaria, with an area of 1560 km^2^, is a consolidated and mature tourist destination with wide international demand. It is one of the islands with the largest population and capacity for accommodation options, attracting 4,189,015 tourists in 2019 to a region with 852,231 inhabitants (ISTAC, 2022). Tourism activity is mainly concentrated in the south of the island through a succession of coastal settlements that have substantially transformed environmental values (Ferrer et al., 2017). In addition to the environmental impacts, a floating population overload on the resident population is an indicator of the potential risks that could occur during situations of extreme precipitation. Moreover, the level of risk is accentuated by its location in the outermost region, which is too remote and isolated to receive immediate foreign aid.

The tourist urbanization in south Gran Canaria began from the beach of San Agustín in the 1960s, when the first urban settlement for mass tourism came up. After the urbanization of the coastal strip, the following decades saw it grow inland with increasing complexity at the orographic and morphological levels. San Agustín is illustrative of tourist urbanization along hillsides with buildings constructed on steep slopes, some very close to the ravines and gorges that cut across the development. It represents an urbanization model that is repeated in later tourist settlements of Gran Canaria such as Patalavaca, Puerto Rico, Amadores or Taurito Beach. A very important part of the urbanized area is located on very pronounced relief areas, approximately between 25 and 75 m of altitude, with an average slope of 27%, which in some cases reach 45%, or even 140% at the most critical points of the island’s topography.

A good part of the tourist complexes here has been developed as groups of low-rise bungalows, although in other cases, hotel typologies and apartment buildings were chosen. However, the abundant irregularities of the relief led to the frequent adoption of accommodation solutions at different levels with the use of terraces or stepped platforms with stairs, walls of a certain height, dismounts and slopes, etc.

**Fig. 1:**
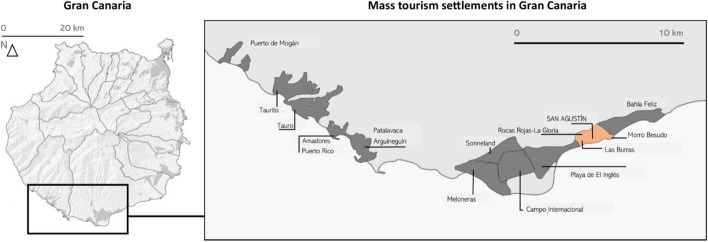
San Agustín in the context of south Gran Canaria. Source: Compiled by the authors

The study area coincides for the most part with the delimitation established by the Special Territorial Plan for Island Tourism Management of Gran Canaria (PTEOTI-GC) that considers several sectors, the central part of San Agustín and Morro Besudo, Las Burras to the east, Playa del Águila to the west and Rocas Rojas–La Gloria up in the hills (Díaz de Aguilar & Garrido, 2013). The delimited area as a whole represents an area of 126.45 hectares, known generically as San Agustín, with an estimated 7450 tourist places (Fig. 1). All this allows us to understand the quantitative importance of the area as a case study, which adds to the complexity described in terms of orography and urban morphology.

The orographic difficulties and the scarcity of space for tourism development entail a contingency urban planning, with urban plots that are unsuitable to respond to the demands of tourist destinations (De Castro, 2021). This poorly planned expansion has often occurred on environments of difficult ecological balance, positioning tourist development on areas susceptible to damage by natural events. Among the main risks identified in the San Agustín area are rockfalls and landslides from surface runoff due to extreme precipitation. These events present San Agustín as an ideal case study for understanding the extreme impacts on mass tourism locations, which may also be exacerbated by climate change. Thus, the floods due to extreme rainfall in San Agustín were the result of a combination of various factors similar to those of other urbanized tourist locations built along hillsides.

## Research aims and methodology

Despite the absence of seasonality in tourist activity in the Canary Islands, all activities peak precisely in the winters, coinciding with a greater probability of rains, as mentioned earlier. Due to its mainly rugged terrain, the south of the island of Gran Canaria is home to several tourist settlements built along the ravines and high slopes. This urbanization pattern is vulnerable to considerable risk from the unforeseen behavior of slopes in this terrain. In addition to floods, extreme rainfall is also among the main causes of landslides on unstable slopes. The analysis of this risk will allow the evaluation of one of the most critical aspects of such settlements.

The vulnerability of a region, understood as a propensity to suffer damage, depends not only on the predisposition of the natural environment to be transformed, but also on the changes introduced during the process of urban development (Cardona, 2003). San Agustín, thus, serves to test the model of tourist urbanization along hillsides, indicating its fragility from the consequences of climate change. It has been especially chosen to demonstrate its vulnerability to extreme rainfall in relation to the specific analysis of the risk of slope dynamics and its possible repercussions in physical terms. Further, owing to the importance of receiving assistance and for evacuation in emergency situations, the problem of accessibility has also been identified for a location like San Agustín that is entirely dependent on road mobility.

This study also points out possible alternatives for risk reduction in the face of such disasters. In this sense, attention should be paid not only to buildings, but also to public spaces as a resource for emergency situations, since they can serve as a reference and guidance for people during the evacuation process (Berroeta et al., 2016). Thus, corrective measures will be proposed to minimize the existing risk, referring to the redesign of both infrastructure and accessibility elements and some private collective spaces.

The Spatial Information System of the Canary Islands (Gobierno de Canarias, 2022) has been used for the analysis, wherein the Spatial Data Infrastructure includes the digital tool, RiesgoMap, with maps of risks associated with climate change. RiesgoMap can provide cartographic information on ‘slope dynamic’ for the study area, as the main risk from extreme rainfall. Based on this information, and through urban analysis supported by digital cartography, orthophotographs and fieldwork, a detailed cartography has been developed for San Agustín, suitable for identifying certain physical elements that could be directly or indirectly affected by an extreme weather event.

The ‘slope dynamic’ risk on the planimetry of San Agustín has been carried out both at a scale of 1:5000 and 1:1000. During the elaboration of the cartography, the elements exposed in sectors of low, medium and high risk, related to the behavior of the terrain, have been identified. On the one hand, retaining walls have been inventoried, differentiating those over 3 m high since their stability can be seriously affected in case of extreme rainfall. This is because the presence of high levels of humidity in the ground considerably alters their consistency and density, among other factors. On the other hand, natural slopes between 50 and 100% have been identified, differentiating them from those that exceed this inclination due to their greater instability in situations of torrential rains. All natural walls and slopes that pose a high risk for adjoining buildings and other built spaces have also been graphically indicated.

This methodological procedure also helps to reduce an area’s vulnerability to extreme rainfall in terms of accessibility. The importance of accessibility for providing assistance and evacuation in emergency situations must be taken into account as well. Hence, sections of the road infrastructure within the medium- and high-risk sectors have also been identified. These are specific sections that would be seriously compromised in the event of extreme rainfall. Thereafter, a series of cuts or interruptions have been assumed at different points of the roads, coinciding with these sectors of medium and high risk. These cuts help to test the effectiveness of the road system in emergency situations, as well as to verify its vulnerability. It makes it possible to study whether certain urban sectors could be easily evacuated or would be left isolated. It also allows the evaluation of the exposed area and the number of accommodations that would be left isolated within each sector. The volume of tourist accommodation exposed to extreme weather events has been calculated on the basis of data from the cadastral information for the Cartography and Maps of Gran Canaria (Cabildo de Gran Canaria, 2022). For all this, a specific cartography has also been elaborated at a scale of 1:5000 through fieldwork and graphic analysis on digital planimetry.

Once the at-risk areas have been identified, some alternatives may be proposed to minimize the vulnerability of the area. On the one hand, the study considers a series of routes aimed at improving accessibility conditions to minimize scenarios that could pose the greatest risk in cases of extreme rainfall. On the other hand, some private collective spaces are recognized in areas with a low, very low or risk-free level, to improve the possibilities of assistance and evacuation in case of emergency. In fact, the potential risk of loss of human lives along with the undesirable alteration of heritage and landscape values could deeply question the attractiveness of a tourist destination as a result of such extreme weather events. The proposed methodology addresses this theme from the urban planning discipline and assesses its direct impact on the physical environment and inhabited space.

## Results

### Extreme rainfall in San Agustín

Tourism activity and physical transformations associated with the urban development of this productive sector often cause changes in the morphology of the coast and in the relief that tends to increase its vulnerability to the effects of climate change (Campos & Puig, 2017). These transformations are almost always evident in the coastal profile, but are not usually sufficiently evaluated in other areas of the relief. Heavy rainfall can lead not only to surface runoff, but also to the displacement of considerable land masses from sloping terrain (Milanés et al., 2017). In a very irregular orographically area such as San Agustín, this can pose a significant risk with potential consequences on material elements as well as on visitors and residents alike.

**Fig. 2:**
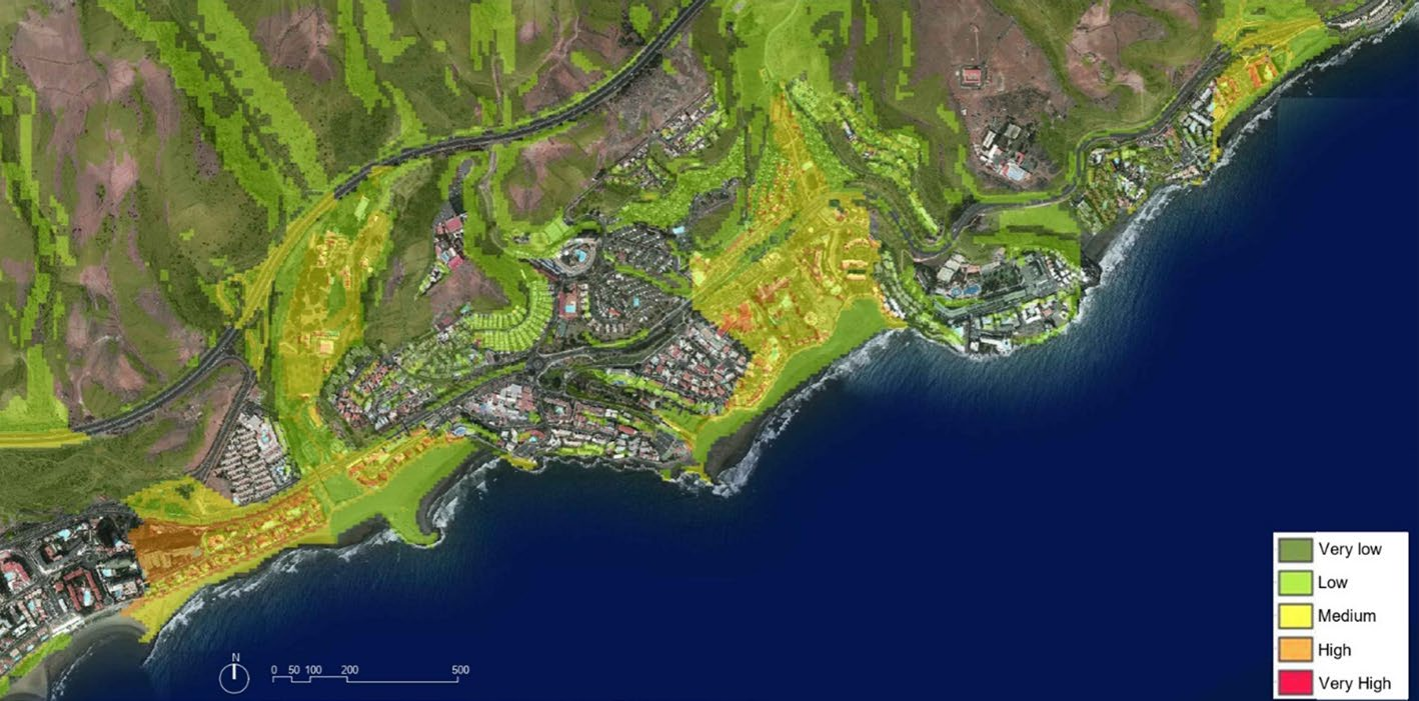
San Agustín: risk due to slope dynamics on orthophotograph. Source: The authors, based on RiesgoMap (Gobierno de Canarias, 2022)

The ‘slope dynamic’ map from RiesgoMap (Gobierno de Canarias, 2022) classifies risk levels into five categories—from very low to very high. The cartographic overlay of this information on planimetry and on the orthophotograph of the same area makes is possible to visualize the exposure of the different levels of risk on the areas considered (Fig. 2). The remarkable levels of risk appear concentrated in the area near the main ravines, especially in those closest to the channels. It tends to increase and widen in areas of lower elevation, but this is where most of the urbanized area is concentrated by the demand characteristic of the ‘sun and beach’ tourism principle. Paradoxically, most areas without risk or very low risk are currently undeveloped.

**Fig. 3:**
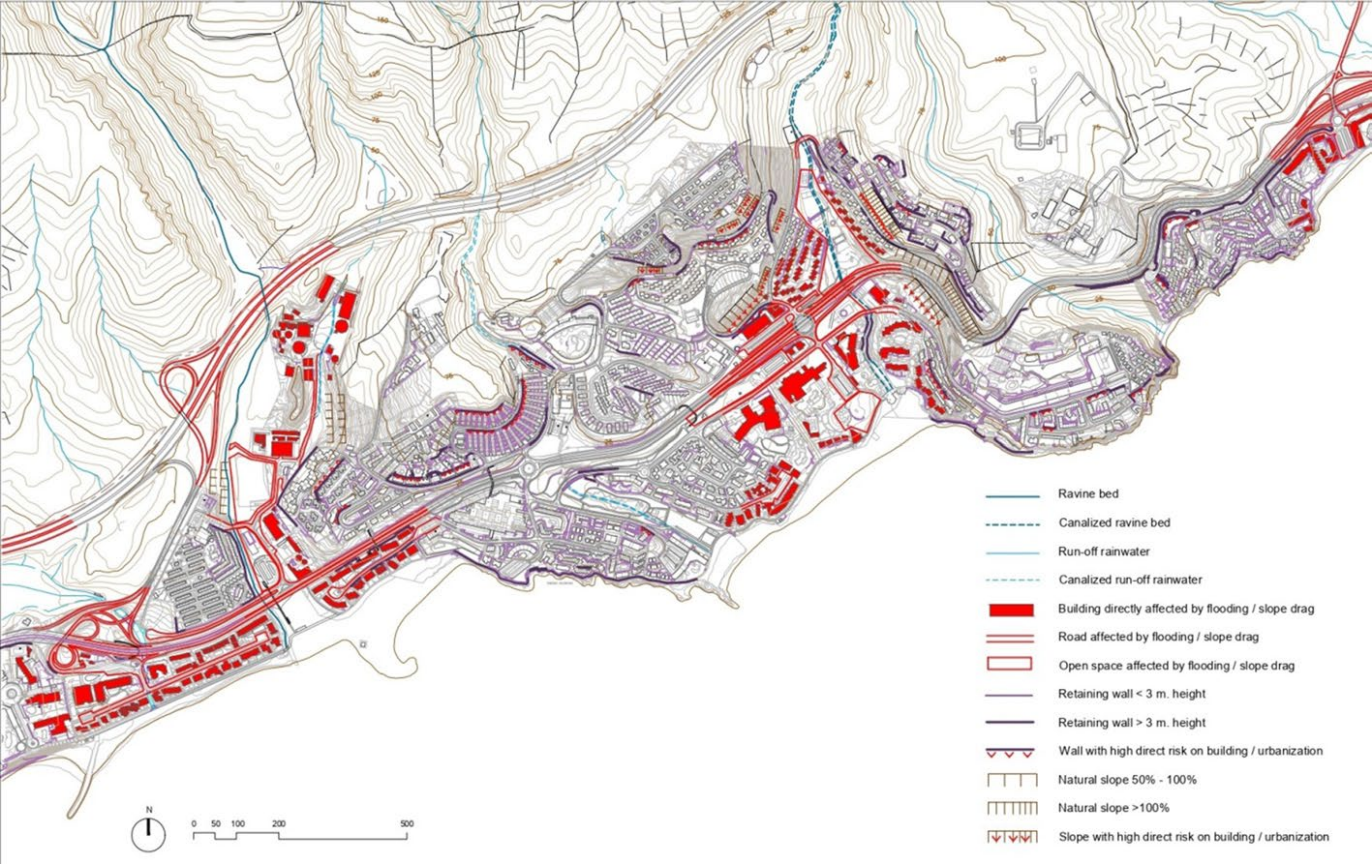
Elements exposed to extreme rainfall and slope dynamics. Source: The authors (P. Ley & Ó. de Castro)

By superimposing these levels of risk on the planimetry of the San Agustín area, all the exposed physical elements can be identified in detail. This provides a detailed cartography suitable for identifying all urban and architectural elements that could be directly or indirectly compromised. As a result, the study has identified all exposed surfaces and elements, as well as containment systems through walls and slopes that represent a significant risk (Fig. 3). In case of extreme rainfall, the instability of these walls and landslides along these slopes can cause significant material damage on buildings, roads and open public spaces. Tourists and visitors could be seriously affected if these areas are not evacuated early and adequately. In addition to an accurate cartographic representation, this analysis helps to quantify the elements that can be seriously compromised during an extreme weather event (Table 1).

**Table 1:** Quantification of exposed physical elements

Total area directly exposed (built-up surface)	73,077 m^2^
Total surface area exposed (open spaces)	60,376 m^2^
Total length of directly exposed road tracks	9190 m
Total length of retaining walls < 3 m. height	33,553 m
Total length of retaining walls > 3 m. height	9615 m
Total length of retaining walls > 3 m. height with direct risk on buildings and other living spaces	4074 m
Total front of natural slopes with slope > 100%	384 m
Total front of natural slopes with slope > 100% with direct risk on buildings and other living spaces	227 m

*Source* Compiled by the authors

These data indicate the quantitative importance acquired by certain physical elements in the face of extreme rainfall and landslide risks. Most of these elements are mainly concentrated in the lower elevations around the main channels of the ravines. On the other hand, a good part of the retaining walls is located in the most orographically complex areas, due to the need to adapt to the conditions of the terrain. Quantifying retaining walls of more than three meters and slopes at a greater than 100% inclination and specifying those with direct risk on buildings and habitable open spaces also help to identify other very significant indicators about the vulnerability of the area.

### Accessibility against the risk of extreme precipitation

**Fig. 4:**
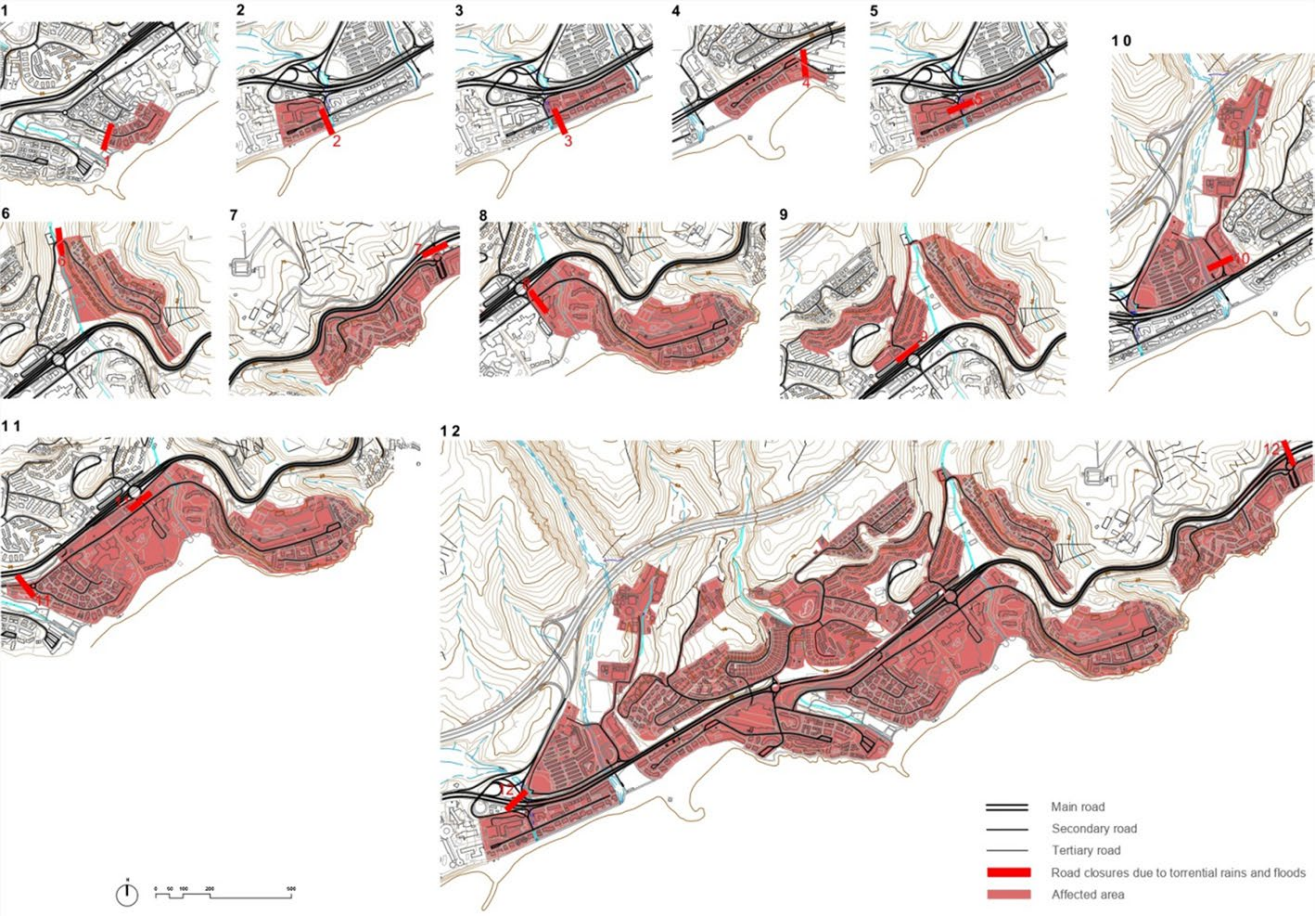
Possible scenarios combining accessibility—extreme rainfall—landslides. Source: The authors (P. Ley & Ó. de Castro)

The cartography on exposed physical elements made it possible to identify those sections of the road included within the sectors of medium and high risk in San Agustín. These include specific intervals or points on the road that would be seriously compromised in the event of a catastrophe due to extreme rainfall and landslides. Thereafter, a series of cuts have been assumed at different points along this route, which coincide with the areas identified by their considerable level of risk. They are different scenarios of cuts or interruptions to test the effectiveness of the road system in emergency situations, or even to verify its vulnerability in catastrophic situations owing to extreme rainfall. The study considers twelve possible situations of cuts (Fig. 4) and identifies important sectors that can be isolated. For each of these twelve cuts, the exposed area has been calculated, as well as the number of accommodations that would become isolated in each sector. The analysis of this casuistry allows us to measure the magnitude that could be achieved in each of these scenarios (see Table 2).

**Table 2:** Quantification of the resulting sectors for each scenario

Road cuts	Surface area per road cuts (m^2^)	Nº of tourist places exposed	Nº of residential places exposed	Total number of accommodation places exposed
Cut 1	19,951	311	0	311
Cut 2	23,711	981	74	1055
Cut 3	30,415	348	118	466
Cut 4	31,421	840	158	998
Cut 5	55,093	1329	192	1521
Cut 6	88,359	160	96	256
Cut 7	93,718	1729	807	2536
Cut 8	165,810	1258	1814	3072
Cut 9	182,627	389	160	549
Cut 10	190,924	725	0	725
Cut 11	306,202	3287	2009	5296
Cut 12	1,237,105	7129	3560	10,689

*Source* Compiled by the authors

Thus, among the scenarios considered, there are sectors of relatively smaller dimensions (Cuts 1 to 4) than others of medium size that are already worrying (Cuts 5 to 10) or even really alarming (between approximately 200,000–1,200,000 m^2^, where more than 10,000 accommodations could become exposed to weather events). Although situations with more serious consequences respond to less likely situations, such as a scenario with two simultaneous road cuts, such hypotheses should not be ruled out in a risk assessment. In addition, situations of immediately lower magnitudes, classified as very worrying, are more likely and could isolate important sectors for immediate evacuation or assistance in an emergency. In any case, the analysis demonstrates the high level of vulnerability registered by the San Agustín road system, severely questioning its effectiveness in the case of extreme rainfall and landslides. Moreover, such a level of fragility occurs within an outermost island environment, characterized by the isolation and dependence inherent in such a geographical context.

## Discussion

Climate change vulnerability analysis is essential for urban planning and sustainable management. But despite the serious risk posed by climate change to mass tourist destinations, such risks are usually not taken into account in their planning processes (Campos & Puig, 2017). The construction of tourist cities involves introducing changes in the original environment by superimposing new structures, forms and uses. Therefore, the vulnerability of coastal and island territories must be weighed from the angle of interaction between the natural and the urban, including risks other than strictly natural (Lavell, 1999).

In this sense, San Agustín houses a considerable number of tourist complexes built in unreasonable positions as far as the natural relief is concerned. This is due to its location on terrain of very steep slope, its excessive proximity to the channels of ravines and runoffs, and its forced position on slopes and coastal cliffs, which allows us to question the convenience of urbanizing and building a good part of this area. In addition, the ravine is channeled with an insufficient section, increasing the chances of overflow in the lower areas of the core. Thus, the accumulation of water in the area near the ravine usually floods part of the commercial establishments located a few meters away and near the beach. The nonexistence or deficient nature of the sewerage network increases the volume of surface runoff that, and causes water to precipitate on to buildings or the road network, dragging earth and solids, sometimes of large volume. As a first effect of this type of process is the collapse of the road network and flooding in hotels and shops, which in turn lack adequate pumping and extraction systems to pump out water. But to this is added the risk of landslide debris and rocks, which considerably increase the vulnerability of the multiple retaining walls and slopes, part of which could overturn directly on to buildings and adjacent open spaces.

This way of urbanizing with little attention to orographic peculiarities is also due to an occupation of the area through tourist complexes that are scarcely related to each other. Because when the master plans were elaborated, they were drafted as a bureaucratic document, as simple zoning without the capacity to articulate the different settlements or their relationship with the coastal area (Ley, 2017). This favored a growth based on partial master plans that was giving rise to a series of urbanizations and tourist complexes with hardly any links between them.

In addition, from the 1960s, coinciding with the establishment of mass tourism in the islands, the process of inclusion of the automobile in Gran Canaria brought with it important physical and social repercussions. This process was characterized by the absence of real alternatives to road mobility and by the weakness of public transport. To this was added a very high percentage of surface area dedicated to road infrastructure compared to other Spanish regions, thus generating a wide network of roads suitable for alleviating the orographic difficulties of the island territory. These roads led to dispersed growth in the second half of the twentieth century, moving from an agrarian economy to a service economy derived from the *tourist boom* of the 1960s (Ley, 2011). Consequently, accessibility to the different areas of the tourist areas in south Gran Canaria is achieved only through road mobility. The movement of tourists, residents, goods or the provision of services of all kinds depends entirely on road infrastructure. This dependence within a small island in an outermost position with respect to Europe already supposes a considerable level of vulnerability to any extreme weather event.

To this, we must add another aspect that will have a considerable impact on the inadequacy of the road system in the San Agustín area. On the one hand, its growth is based on partial plans and urbanizations that are poorly linked with each other. On the other hand, the privacy requirements of tourist accommodation make connectivity with the surrounding urban space not a priority at all. All this has been generating a weak interconnection between the various tourist complexes, which are hooked almost directly to the main roads through a tree system for the most part. This translates into the usual presence of important urbanized sectors with a single entry and exit route that ends in *cul-de-sac*.

In fact, stretches of road seriously compromised in the event of extreme rainfall clearly call into question the efficiency of the road system to ensure accessibility. This is proved by considering a series of cuts or interruptions at critical points of the road, established as different scenarios associated with the risk of extreme rainfall and landslides. The evaluation of the consequences of these cuts through the data related to surface area and number of accommodation places exposed in each of the sectors that would be isolated demonstrates the magnitude of the risk, which in certain situations can be described as potentially alarming.

However, accessibility is a determining factor in enhancing the security of an urban area. Access to transportation and specialized personnel are crucial for providing assistance and evacuation in emergency situations. The efficiency of the road system is often fundamental in this regard. To improve the possibilities of evacuation and assistance in emergency situations, a series of alternatives have been proposed for San Agustín. These alternatives are related to two specific features of tourist urbanization: (a.) the high level of floating population over the resident population and (b.) the relevance of private collective space with respect to conventional public space (Ley, 2017).

San Agustín is identified with a high level of floating population, where accommodations not recognized as tourist places account for only 33% of the total, compared to 67% of beds in the tourist exploitation regime. The ‘non-tourist’ places correspond mainly to second homes for weekends or holidays, preferably in summers, intended for the local population. But many of these places are usually rented during the busiest season through formulas such as holiday homes or similar schemes. Therefore, in winters, the floating population can increase well above the indicated percentage. In addition, in the Canary Islands the areas destined for mass tourism, such as south Gran Canaria, are characterized by short stays. Specifically, the average period of stay in the central area of San Agustín is 8.27 days, while that in adjacent Las Burras is slightly less with 7.85 days on average (TGC, 2020).

This is how the high percentage of the island’s floating population and their short durations of stay are usually decisive in the event of emergency situations from extreme weather events. Logically, most tourists lack a precise knowledge of both the urban and the architectural environment in which they enjoy their brief holiday experience. This makes them unaware of the exit routes or connections with other areas, including difficulties in choosing the most efficient options, and, in short, makes them prone to experience some disorientation in emergency situations.

**Fig. 5:**
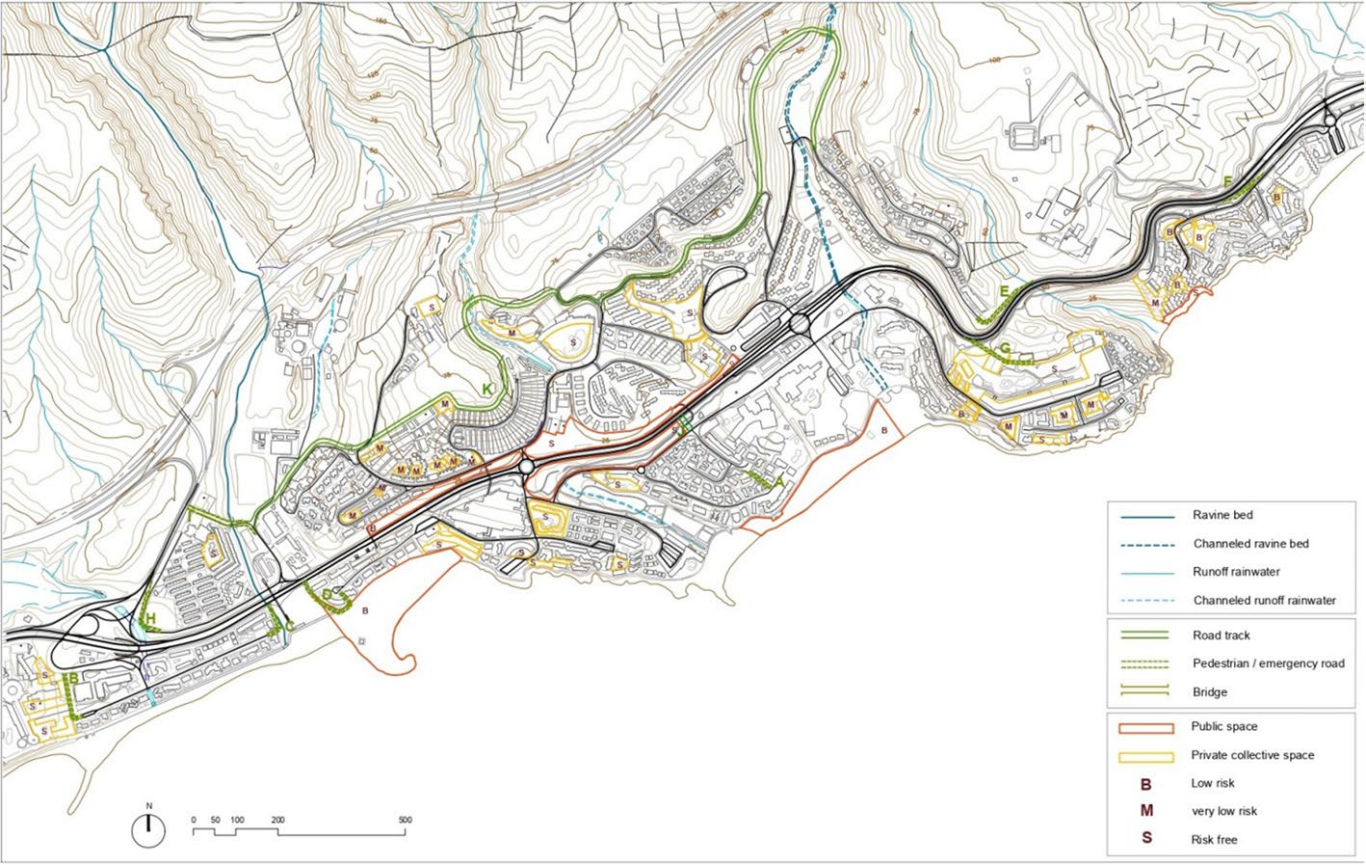
Proposal of alternatives. Vulnerability combining accessibility—extreme rainfall—landslides. Source: The authors (P. Ley & Ó. de Castro)

Furthermore, the road system in San Agustín is marked by its arborescent character, through a disorderly plot with frequent loops in *cul-de-sac*. This almost labyrinthine and unresolved character of the road further propitiates the disorientation of tourists. Therefore, all this would undoubtedly make it difficult to evacuate them rapidly or even to quickly send assistance to the right places. Improving the possibilities of evacuation and care in emergency situations means reducing the vulnerability of San Agustín in terms of accessibility. To this end, alternatives are needed to improve the low efficiency of its road system. Based on the analysis carried out in this study, a series of routes are proposed to improve the conditions of general accessibility and connectivity between different parts of San Agustín. The improvement strategy would be to reduce the number of roads at *cul-de-sac*, particularly those that would pose the greatest risk in emergency situations (Fig. 5).

The accessibility study allows to establish concrete alternatives to considerably minimize the risk associated with the possible road cuts previously detected. The proposed road connections are specific responses to each of the twelve scenarios studied. For the design of each proposed connection, the type of road (road track or pedestrian/emergency), its average slope, and the number of properties exposed (with the necessary percentage of open spaces to be obtained through specific urban management tools) are analyzed. Also defined are the other complementary characteristics and level of risk on which each of the proposed alternatives is located (see Table 3).

**Table 3:** Proposed accessibility alternatives

Cut	Connection proposal	Type of road	Medium slope	Exposed plots	Other features	Risk level
1	A	Pedestrian (road track)	8%	2 plots (6%)	Widening of pedestrian path	Medium–low
25	B	Pedestrian (road track)	1%	1 plot (4%)	Widening of pedestrian path	Middle
35	C	Pedestrian (road track)	4%	1 plot (6%)	Widening of pedestrian path	Middle
4	D	Pedestrian (road track)	2%	1 plot (3%)	Widening of pedestrian path	Middle
69	E	Pedestrian (road track)	5%	–	New track	No risk
7	F	Pedestrian (road track)	10%	–	New track	Very low
811	G	Pedestrian (road track)	4%	1 plot (4%)	New track	Very low
10	H	Pedestrian (road track)	7%	–	New track	Middle
10	I	Pedestrian (road track)	1%	–	New track	Very low
11	J	Road track	1%	–	Treatment of existing road track	No risk
12	K	Pedestrian/Road track	4–12%	–	Widening of pedestrian paths and new cornice road	Low–very low

*Source* Compiled by the authors

The proposed road connections would provide greater safety for tourists as well as other added roads. By considerably reducing the number of roads in *cul-de-sac*, its mesh character is enhanced, clarifying the formal and functional structure of the road system of San Agustín. All this would make it possible to boost the network of pedestrian paths through new routes, as well as to introduce a cornice road to improve road traffic in the upper part, also creating a pedestrian route with views over the coastal landscape.

On the other hand, in addition to the high level of floating population, another characteristic feature of tourist urbanization is the weakening of the traditional role of public spaces. This is due to the fact that much of the social activity in these settlements tends to move toward certain private spaces with collective meaning. Public spaces such as shopping centers or leisure areas of each accommodation establishment assume a determining social role (Ley, 2017). There are some public spaces such as promenades that still have some relevance. But the preponderant value of private collective space is a feature of tourist urbanization, especially interesting to consider while forecasting against risks from extreme weather events. The option of considering private collective spaces is compatible with conventional public spaces as resources in the face of extreme events.

Public spaces can serve as a reference point and guidance for people, both during and after disasters (Montejano-Castillo & Moreno-Villanueva, 2017). They can also serve to reactivate certain activities after catastrophic situations, especially when living conditions cannot be restored very quickly. Private collective spaces, however, can also be considered safe to serve as meeting points and attention in emergency situations. To reduce the vulnerability of San Agustín, it is necessary to identify a series of public and private spaces that are safe and that can be added to the proposed routes. The aim is to show a network of safe spaces and routes that can be recognized by tourists and residents through different available means (signage, inclusion in mobile applications) and that can become part of evacuation plans (Fig. 5). The surfaces of public and private collective spaces available in relation to the different levels of risk allow quantifying the magnitude of risk corresponding to each situation (Table 4).

**Table 4:** Quantification of public and private collective spaces

Types of collective spaces	Low risk	Very low risk	No risk	Surface area
Private collective spaces	8456 m^2^	11,228 m^2^	73,969 m^2^	93,653 m^2^
Public space	69,506 m^2^	–	33,236 m^2^	102,742 m^2^

*Source* Compiled by the authors

The total number of public spaces and private collective spaces with an acceptable level of risk (196,395 m^2^) allows us to verify their enormous potential to cope with assistance and evacuation in case of extreme rainfall. It also notes the quantitative importance of private collective space (48% of the total), as well as the relevance of public and private spaces in areas without any risk (107,205 m^2^). The identification of public and private spaces suitable for ensuring care and evacuation in emergency situations thus becomes an important strategy for adaptation to climate change. All this serves to configure a network of spaces and safe routes recognizable for tourists and residents. Therefore, in addition to public spaces, private collective spaces also have considerable potential to collaborate in reducing the vulnerability of mass tourist settlements in the event of extreme rainfall and landslides.

## Conclusion

Climate change represents a considerable risk for coastal developments linked to mass tourism, more for those located in geographically isolated and highly dependent contexts such as the Canary Islands. It is, therefore, necessary to address the specific risks of tourist urbanization on small islands such as Gran Canaria, as well as to reduce their levels of vulnerability.

San Agustín was urbanized without clear morphological patterns and without a coherent response to the complexity of its orography. Several buildings and tourist complexes were located in inadequate positions due to their proximity to ravines and slopes, as well as for occupying spaces with an excessive slope. This translates to many urban and architectural elements being exposed to certain risks such as extreme rainfall and landslides, which are expected to increase as a result of climate change. In addition, its urban growth based on relatively autonomous operations, and on a tree road structure, has led to physical and functional segregation between various tourist complexes.

In case of extreme rainfall, the highest concentration of medium- and high-risk areas coincides precisely with the most intensely urbanized areas of San Agustín. The present analysis highlights the quantitative importance of surface area occupied by buildings, roads and open spaces subject to such risks. The considerable number of high-risk walls and slopes near inhabited spaces is also evident. Such built elements are concentrated mainly in the lower elevations around the channels of the ravines. This wide diversity of especially unsafe urban and architectural elements indicates the vulnerability of the area to considerable material damage with possible loss of human lives in the face of extreme events.

On the other hand, the importance of accessibility in emergency situations means that the absolute dependence on road mobility in San Agustín already shows a first level of vulnerability. Coupled with a tree road system, this is incapable of effectively solving the problem of accessibility to various tourist complexes. Thus, the detection of various critical points along the road system clearly demonstrates its high level of vulnerability. This shows a high probability that large urban sectors with a considerable number of tourists could be isolated in case of extreme rainfall.

It has also shown how reducing risks from extreme precipitation can be implemented through various corrective measures. This can be achieved by redesigning the road system, enhancing its mesh character to minimize the autonomous nature of the different sectors and tourist complexes, as well as by evaluating not only the built environment but also public and private collective spaces as potential resources in emergency situations. Developing this network of safe, public and private spaces, as well as their connections, will promote the resilience of the area to risk from climate change.

This case study, therefore, serves to verify the usefulness of the proposed methodology. San Agustín is a representative case for certain situations based on the model of tourist urbanization along hillsides. This form of urban development for mass tourism highlights its vulnerability in the event of extreme rainfall. But the methodology shows how it is possible to propose some alternatives related to the problem of tourist urbanization along hillsides against certain risks from extreme weather events. Further, these criteria can be incorporated into urban planning strategies to promote the resilience of island destinations for mass tourism in the face of climate change.

## Declarations

### Data availability

Data will be made available on reasonable request.
